# TAVI Under Pressure: Intra-balloon Pressure Profiles During Balloon-Expandable TAVR—First Data from a Feasibility Study

**DOI:** 10.1007/s12265-022-10281-6

**Published:** 2022-06-06

**Authors:** Timothée Noterdaeme, Nikolaus Marx, Hans Theiss, Martin Orban, Daniel Roden, Steffen Massberg, Daniel Braun

**Affiliations:** 1 Department of Cardiology & Intensive Care Medicine, University Hospital Aachen, RWTH Aachen University, Pauwelstrasse 30, 52074 Aachen, Germany; 2 Munich University Center, Ludwig-Maximilian University, and German Centre for Cardiovascular Research (DZHK), Partner Site Munich Heart Alliance, Munich, Germany

**Keywords:** Transcatheter aortic valve implantation, Complications, Paravalvular leakage, Underexpansion, Pressure

## Abstract

Our study investigated the feasibility to measure pressure profiles inside the inflation balloon during direct implantation of Edwards Sapien 3 ultra-prostheses using an additional syringe with a digital pressure read-out. Pressure profiles of 15 patients for 26 mm valve size were analyzed. Uniform patterns were found for 5 patients similar to those of previously acquired in vitro curves. 10 patients showed strikingly different pressure profiles compared to the above-mentioned group, marked by an earlier pressure increase, single or multiple pressure drops or higher overall pressure. Measuring the percentage of under-expansion of the prostheses, using calibrated angiographic projections revealed a significant difference between both groups. Our data raises the hypothesis that the acquisition of pressure profiles might help to better understand not only the implantation procedure itself but also the highly individual patient-device interaction, offering new information and a new perspective on optimization of TAVR implantation in the future.

Balloon-expandable transcatheter aortic valves have shown excellent results, even surpassing surgical aortic valve replacement in short-term observation [1]. Yet, the known shortcomings of PVL, pacemaker implantation, and valve deterioration remain a concern. Interventional cardiologists are accustomed to pressure-guided implantation strategies for coronary stents, yet in balloon-expandable TAVR fixed inflation volume are used and the applied pressure is judged by intuition and manual feeling. A recent study investigated the feasibility of pressure-guided implantation [2] using a predefined value. However, the role of the exerted pressure and how it is applied during the implantation have never been studied, even though it might be one of the biggest contributors and pivotal for achieving desirable long-term results. We therefore investigated the feasibility to measure pressure profiles inside the inflation balloon during direct implantation of Edwards Sapien 3 ultra-prostheses. Using an additional syringe with a digital pressure read-out (Merit Medical Intellisystem) connected as “innocent bystander” to the 3-way stop-cock, datapoints were collected every 10 ms. Recordings started with the initiation of rapid pacing and lasted for 20 s. The implanters were blinded to the recording during the implantation as to not affect their decision-making. After exclusion of patients with bicuspid anatomy, valve in valve implantation, volume manipulation, or predilatation, pressure profiles of 15 patients for 26 mm valve size were analyzed. Pressure profiles and main clinical characteristics are summarized in Fig. 1. The profiles, adjusted for different inflation speeds via interpolation and using the point where the syringe has been fully emptied as reference, could be grouped into two categories. For 5 patients, a uniform pattern emerged (dashed lines) resembling those of unconstrained curves acquired in a previous experiment using an automatic inflation device in non-calcified xenografts [3]. In contrast to this “unconstrained” group, 10 patients showed strikingly different pressure profiles, marked by an earlier pressure increase, single or multiple pressure drops, or higher overall pressure (solid lines). Even though parameters like Agatston score (2971 vs 3427; *p* = 0,71), peak-pressure (7305 vs 7423 mbar; *p* = 0,75), annulus surface (480 vs 506 mm^2^; *p* = 0,89), and perimeter (79 vs 81 mm; *p* = 0,80) did not differ, we found a statistically significant difference in the percentage of under-expansion (diameter at commissure level/max theoretical achievable diameter) of the prostheses (4.9 vs 10.4%; *p* = 0.02). Measurements were made using a standardized iso-center calibrated angiographic projections and a pigtail as size reference. The pressure profiles and peak-pressures are reflecting the heterogenic pathology and patient population of aortic stenosis, where a multitude of factors influence the outcome. Like coronary stents, where malaposition affects long-term results, under-expansion of the aortic valve stent [4, 5] might impede hemodynamic performance thus contributing to earlier valve failure and PVL. Further studies are warranted to assess the correlation of those adverse events with deviation from the “unconstrained line” of implantation as well as with the maximum exerted pressure, as suboptimal results bear a heavier toll in the population of low-risk patients.

**Fig. 1:**
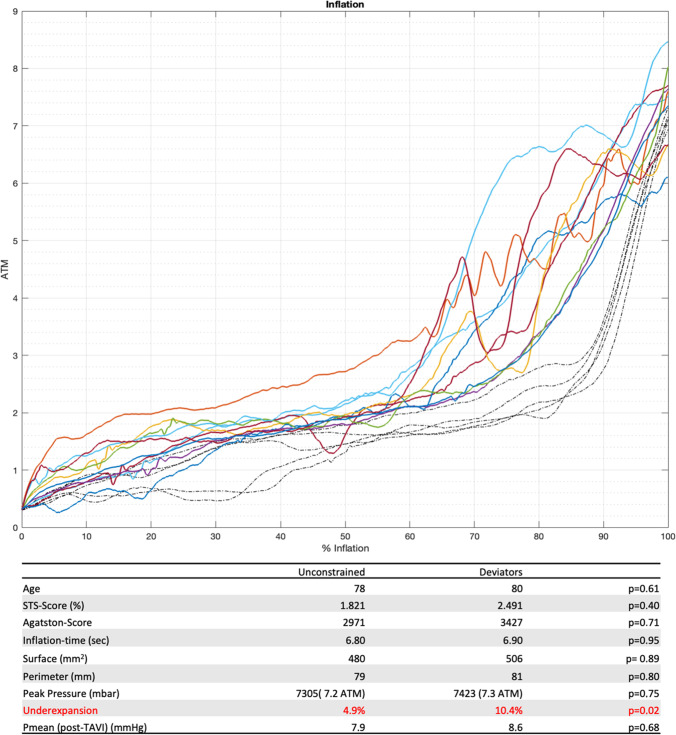
Pressure-profiles and main clinical characteristics. Profiles for “unconstrained” are represented in dashed lines whilst “Deviators” are in solid lines. The *x*-axis expresses the percentage of completed inflation. Below summarized clinical characteristics of both groups

Implantation via fixed volume inflation or judging the amount of volume manipulation manually might prove inadequate to address these points in such a heterogenic pathology, where the amount of calcification is not the only determining factor of stenotic severity and where other factors contribute to maximum inflation pressure.

As demonstrated by the pressure profiles in our study, using solely the exerted pressure to guide the implantation would have led to suboptimal results. An inflation pressure caped at 5.5 ATM for 26 mm valves, as proposed by Snir et al. [2], would have caused an incomplete inflation with only 70–80% of the volume applied in most of our patients. Using higher cut-off values might on the other hand increase the likelihood of annular rupture depending on the anatomical conditions.

A high effective orifice area remains a hallmark for any valve replacement. As the diameter (d) and the maximal effective orifice area have a quadratic correlation ($A=\pi {(\frac{d}{2})}^{2}$), emphasis should be to maximize the largest achievable deployment. Limiting the exerted pressure to a predefined value without taking into consideration how much volume has been applied may therefore prove equally inadequate as fixed volume inflation alone with either the risk of under-expansion or on the opposite annular rupture.

Our study for the first time describes different pressure/time profiles during TAVR, suggesting that measurement of pressure profile during implantation is feasible. Our data raises the hypothesis that the acquisition of pressure profiles might help to better understand not only the implantation procedure itself but also the highly individual patient-device interaction, offering additional information and a new perspective on optimization of TAVR. Further studies are warranted to investigate whether such profiles and their interpretation can identify and correlate with acute clinical events such as annular rupture as well as events impeding long-term clinical outcome such as PVL, pacemaker-implantation, or hemodynamic performance affecting long-term valve durability.

## Abbreviations

TAVR: Transcatheter aortic valve replacement
PVL: Paravalvular leakage

## Declarations

### Animal Studies

No animal studies were carried out by the authors for this article
